# Mucinous cystadenocarcinoma of the breast: a new entity with broad differentials—a case report

**DOI:** 10.1186/s43046-022-00112-9

**Published:** 2022-02-28

**Authors:** Kanwalpreet Kaur, Ashini Shah, Jahnvi Gandhi, Priti Trivedi

**Affiliations:** Department of Oncopathology, Gujarat Cancer and Research Institute, B/113 first floor, new building, Ahmedabad, India

**Keywords:** Breast, Immunohistochemistry, Primary mucinous cystadenocarcinoma, Case report

## Abstract

**Background:**

Mucinous cystadenocarcinoma is a rare and recently described primary breast cancer with strikingly similar histomorphology to ovarian, pancreatic, and gastrointestinal counterparts. The diagnosis cannot be made until the metastatic lesion is ruled out.

**Case presentation:**

We are reporting the case of a 65-year-old woman with primary mucinous cystadenocarcinoma of the breast while exploring clinicopathological features and approach to diagnosis. Though the immunohistochemistry panel of CK7, CK20, CDX2, SATB2, PAX8, mammoglobin, and GATA3 plays a crucial role in ruling out metastasis but aberrant CK20 positivity was seen in our case, the final diagnosis was made after a complete radiological workup. We also noted strong membranous HER2-protein expression and HER2-gene amplification by fluorescence in situ hybridization while in literature this tumor is reported to show mainly triple-negative basal type immunophenotype.

**Conclusion:**

A combined clinic-radio-immunohistochemical approach is essential to make a diagnosis of primary mucinous cystadenocarcinoma.

## Background

Mucinous cystadenocarcinoma (MCA) is an extremely rare variant of invasive breast carcinoma characterized by cystic structures lined by columnar cells with abundant intracellular and extracellular mucin. It is histologically similar to pancreatobiliary or ovarian mucinous cystadenocarcinoma. It has been recognized as a distinct entity in the recent 5th edition of WHO classification of tumors of the breast, 2019 [1].

To the best of our knowledge, less than 30 cases have been reported till date. The aim of this study is to enrich the literature with another case diagnosed with this new emerging entity and to shed light on its histological and immunohistochemical (IHC) features along with a differential diagnosis.

## Case presentation

**Fig. 1:**
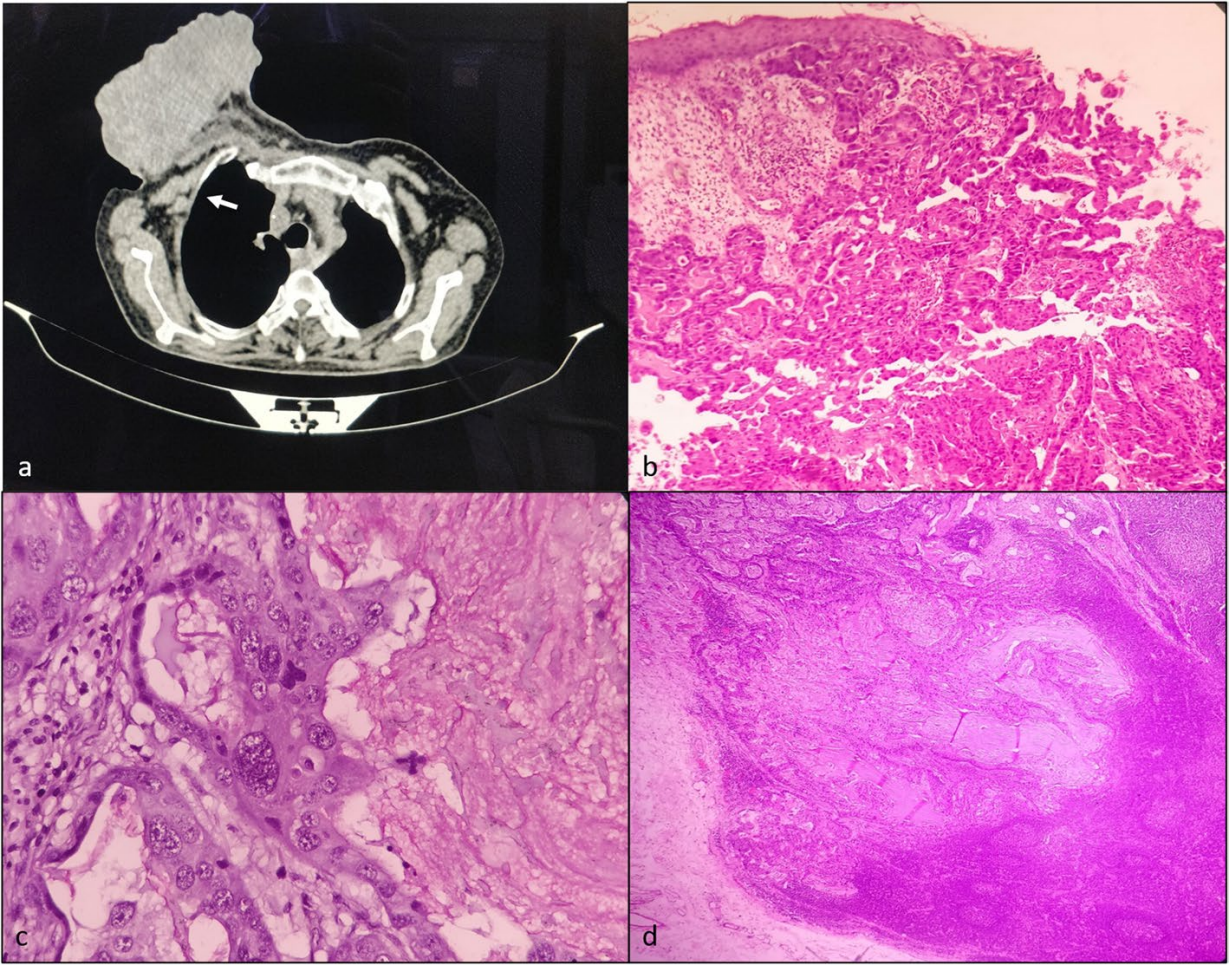
**a** CT thorax showing heterogenous enhancing soft tissue density lesion involving the whole of the right breast with few enlarged lymph nodes with the thick cortex (arrow). **b** Tumor is ulcerating skin (H&E, 10×). **c** Nottingham nuclear grade 3 (H&E, 40×). **d** Lymph node metastasis (H&E, 10×)

A 65-year-old woman presented with a lump in the right breast with pain and discharge since 1 year. CT thorax revealed 16 × 11.5 × 6.5 cm^3^ heterogeneously enhancing soft tissue density lesion involving the whole of the right breast along with few well-defined opacities in both lungs suggesting metastasis (Fig. 1a).

Biopsy was reported as invasive ductal carcinoma with extracellular mucin. This was followed by modified radical mastectomy which revealed a tumor of 18 × 11 × 6 cm^3^ involving all four quadrants of the right breast and overlying skin. Grossly, the tumor was nodular, circumscribed, pushing margins with glistening gelatinous cut surface.

Histopathology revealed a multicystic tumor composed of variably sized interconnected mucin-filled cysts lined by tall columnar cells showing stratification and forming papillary excrescences extending into the lumen. Only small clusters were seen floating in the mucin in few cysts. No myoepithelial cells were seen. Marked nuclear pleomorphism, high mitotic activity (>20/10hpf), and large areas of necrosis were noted (Fig. 1c). There was a tiny focus of high nuclear grade ductal carcinoma in situ component with cribriform architecture. One out of 15 axillary lymph nodes showed metastasis with extranodal extension (Fig. 1d).

**Fig. 2:**
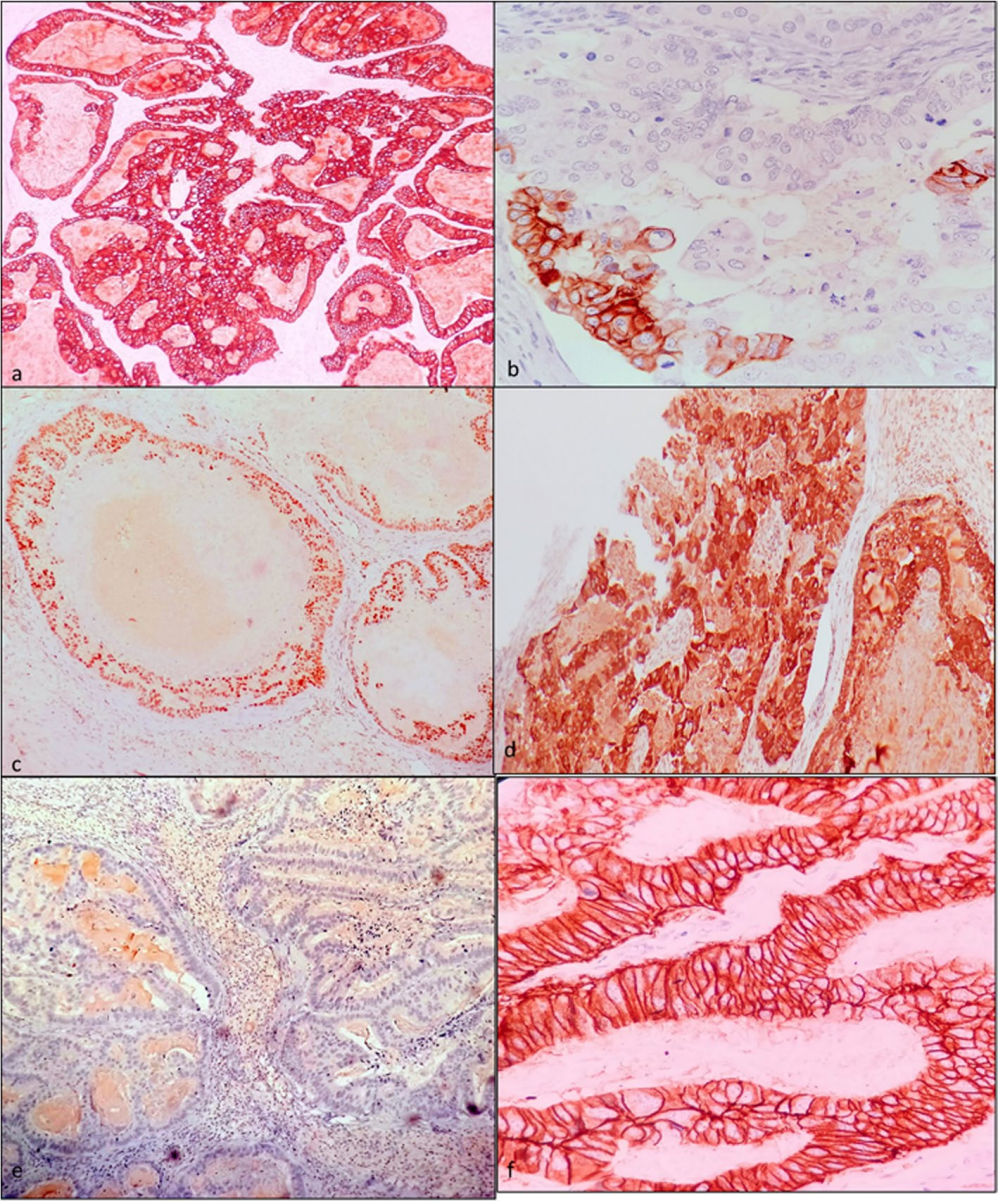
**a** CK 7 membranous positive. **b** Focal CK20 positive. **c** GATA3 nuclear positive. **d** Mammoglobin cytoplasmic positive. **e** p63 reveals the absence of myopeithelial cells. **f** Her2neu:3+

The differential diagnosis based on location and morphology included metastasis versus primary MCA. On performing IHC, tumor cells were diffusely positive for CK7, GATA3, mammoglobin, and MUC1 and focal positive for CK20 while negative for ER, PR, CDX2, SATB2, TTF1, PAX8, WT1, MUC2, and MUC5AC (Fig. 2). p63 revealed the absence of myoepithelial cells. Ki-67 was about 90%. IHC favored primary breast still due to focal CK20 positivity; ultrasound abdomen and PET-CT were performed to rule out any occult primary in the adnexa or gastrointestinal tract. No abnormality was detected; hence on the basis of IHC and radiology, it was considered as primary MCA of the breast and staged as pT4bN1a. Hormonal profile was ER, PR negativity and Her2neu positive (3+) (Fig. 2f). Due to the reported rarity of her2neu positivity, dual-probe FISH for Her2neu was also performed and it showed amplified *Her2neu* with HER2/CEP17 ratio >5 and an average number of *HER2* signals per cell >10. The patient is currently on paclitaxel 260mg since the past 6 months.

## Discussion

Unlike histological counterparts of the ovary and pancreas, MCA of the breast is extremely rare and was first described in 1998 by Koenig and Tavassoli [2].

All the cases of MCA breast have been reported in postmenopausal women with a median age of 61 years (range 41–96 years) and our patient is also a postmenopausal 65 years old [2–12]. Tumor size in our case was very large being 18cm while rest reported size up to 10cm with only a single case of 19cm [2]. All cases described histomorphology similar to that seen in our case. Three cases have reported focal squamous cell carcinoma differentiation and one case high-grade sarcomatoid components [2–12]. MCA of the breast needs to be differentiated from other mucin-producing tumors of the breast. Mucinous carcinoma of the breast does not form cystic structures rather only clusters of epithelial cells suspended in extracellular mucin. Nuclear grade in mucinous carcinoma is low to intermediate while high-grade nuclei have been reported in MCA breast. Mucinous carcinoma of the breast is strongly and diffusely positive for ER and PR positive while MCA is negative [1]. Non-neoplastic mucocele-like lesions show distended mucin-filled ducts but the presence of myopeithelial cells and lack of nuclear atypia helps in the diagnosis [1]. MCA also needs to be distinguished from another rare cystic hypersecretory carcinoma of the breast which also shows multiple variable-sized cystic spaces but contains colloid-like eosinophilic material that often retracts from the epithelium and no intracellular mucin is seen.

MCA of the breast is rare and shares same morphology with ovarian, pancreatic, and/or appendiceal counterparts. Hence, for the diagnosis of primary breast MCA, the ovary, pancreas, and gastrointestinal tract should be excluded first by combing clinical, radiological, and IHC features. The breast is not a common and early metastatic site of MCA in the ovary or GIT, and the patient should have clinical manifestations of the primary lesion and other metastatic sites before the metastatic lesion appears in the breast in most cases. Incidence of metastatic MCA in the breast as the first sign of presentation is extremely rare. The presence of DCIS favors primary over metastasis.

IHC is a powerful tool to distinguish, although a combination of CK7 and CK20 is useful as both primary pancreatic and ovarian MCA are positive for both while breast MCA is negative for CK20. But our case showed focal CK20 positivity and Chen et al. also reported the same [8]. So it is essential to add lineage markers like CDX2, PAX8, GATA3, and GCDFP in the panel.

Most MCA of the breast are triple negative being negative for ER, PR, and HER2. Only 4 cases including the present case were positive for HER2 and FISH was also positive for HER2 [9–11]. Rakici et al. have reported ER positivity [12]. Proliferative index Ki67 has been reported in a wide range of 20.5 to 90% [2–13]. We also reported a high Ki67 index of 90%. Kim et al. reported MUC 5 positivity in intracellular mucin while tumor cells were positive for MUC1 and MUC5 while negative for MUC2 and MUC6 [13]. In our case, tumor cells were only positive for MUC1 while negative for MUC2 and MUC5. Only occasional cells in our case showed intracellular mucin.

The risk of lymph node metastasis is low with apart from the current case, only 4 cases have reported ipsilateral axillary lymph node metastasis and a single case of isolated tumor cells (ITC) [2, 4–7]. No metastasis have been reported in the literature but our case had radiological evidence of lung metastasis at the time of presentation.

Due to the limited number of cases, treatment strategies are still being devised. Most of the previously reported cases underwent partial or radical mastectomy, followed by chemotherapy and radiotherapy. Our patient is also on platinum-based chemotherapy now. Follow-up results ranging from 3 to 46 months indicate that the good prognosis in patients with MCA [3].

## Conclusion

It is necessary to combine clinical, imaging, and IHC to exclude the abnormalities in other organs first before rendering the diagnosis of MCA in the breast. Little is known about the biological behavior, prognosis, and molecular study of MCA of the breast. Therefore, more cases with follow-up data are needed to reveal the biological behavior of this rare tumor.

## Data Availability

Available on request from the corresponding author

## Abbreviations

MCA: Mucinous cystadenocarcinoma
CK: Cytokeratin
CDX2: Caudal-related homeobox gene 2
SATB2: Special AT-rich sequence-binding protein 2
PAX: Paired box gene
HER2: Human epidermal growth factor receptor 2
ER: Estrogen receptor
PR: Progesterone receptor
WHO: World Health Organization
IHC: Immunohistochemistry
MUC: Mucin gene
CT: Computed tomography
PET-CT: Positron emission tomography and computed tomography
